# The New Anthelmintic Tribendimidine is an L-type (Levamisole and Pyrantel) Nicotinic Acetylcholine Receptor Agonist

**DOI:** 10.1371/journal.pntd.0000499

**Published:** 2009-08-11

**Authors:** Yan Hu, Shu-Hua Xiao, Raffi V. Aroian

**Affiliations:** 1 Section of Cell and Developmental Biology, University of California, San Diego, La Jolla, California, United States of America; 2 National Institute of Parasitic Diseases, Chinese Center for Disease Control and Prevention, Shanghai, People's Republic of China; Yale Child Health Research Center, United States of America

## Abstract

**Background:**

Intestinal parasitic nematodes such as hookworms, *Ascaris lumbricoides*, and *Trichuris trichiura* are amongst most prevalent tropical parasites in the world today. Although these parasites cause a tremendous disease burden, we have very few anthelmintic drugs with which to treat them. In the past three decades only one new anthelmintic, tribendimidine, has been developed and taken into human clinical trials. Studies show that tribendimidine is safe and has good clinical activity against *Ascaris* and hookworms. However, little is known about its mechanism of action and potential resistance pathway(s). Such information is important for preventing, detecting, and managing resistance, for safety considerations, and for knowing how to combine tribendimidine with other anthelmintics.

**Methodology/Principal Findings:**

To investigate how tribendimidine works and how resistance to it might develop, we turned to the genetically tractable nematode, *Caenorhabditis elegans*. When exposed to tribendimidine, *C. elegans* hermaphrodites undergo a near immediate loss of motility; longer exposure results in extensive body damage, developmental arrest, reductions in fecundity, and/or death. We performed a forward genetic screen for tribendimidine-resistant mutants and obtained ten resistant alleles that fall into four complementation groups. Intoxication assays, complementation tests, genetic mapping experiments, and sequencing of nucleic acids indicate tribendimidine-resistant mutants are resistant also to levamisole and pyrantel and alter the same genes that mutate to levamisole resistance. Furthermore, we demonstrate that eleven *C. elegans* mutants isolated based on their ability to resist levamisole are also resistant to tribendimidine.

**Conclusions/Significance:**

Our results demonstrate that the mechanism of action of tribendimidine against nematodes is the same as levamisole and pyrantel, namely, tribendimidine is an L-subtype nAChR agonist. Thus, tribendimidine may not be a viable anthelmintic where resistance to levamisole or pyrantel already exists but could productively be used where resistance to benzimidazoles exists or could be combined with this class of anthelmintics.

## Author Summary

Intestinal parasitic nematodes or roundworms infect over 1 billion people in tropical countries. Overall, they are a huge source of morbidity in infected people, including children and pregnant women, and are increasingly being recognized as key poverty-promoting parasites. Despite their importance, few drugs for dealing with them exist. Furthermore, none has optimal efficacy, all can be resisted by the parasites, and, for practical reasons, only one is used for single-dose Mass Drug Administrations (MDAs). There is a dire need for better roundworm drugs (anthelmintics). In the past 30 years, only one anthelmintic, tribendimidine, developed by the Chinese CDC, has entered human clinical trials. Tribendimidine has good single-dose efficacy against some roundworm parasites. However, how tribendimidine works was unknown. Here, using the roundworm *Caenorhabditis elegans* to evolve resistance to tribendimidine in the lab, followed by genetic and molecular testing and cross-resistance drug studies, we demonstrate that tribendimidine is unequivocally in the same drug family as two known anthelmintics, levamisole and pyrantel. These results have important implications for how tribendimidine might be used in MDAs where resistance to current drugs is known or suspected and for how tribendimidine might be combined with other drugs to maximize therapy while minimizing resistance threats.

## Introduction

Thirteen neglected tropical diseases have tremendous impact on the lives of billions of the poorest peoples in the world with an estimated total disease burden of 56.6 million disability-adjusted life years, exceeding that of malaria (46.5 million) and tuberculosis (34.7 million) [1],[2]. These diseases play a major role in keeping infected peoples mired in poverty and in a low socioeconomic state [1],[2]. The top three of these poverty-promoting tropical diseases are caused by intestinal nematodes: ascariasis (caused by *Ascaris lumbricoides*), trichuriasis (caused by *Trichuris trichiura* or whipworm), and hookworm disease (caused by *Necator americanus* and *Acylostoma duodenale*). These parasites (hookworms, *Ascaris*, and *Trichuris* or HAT) are amongst the most common human parasitic infections, with an estimated 576–740 million people infected with hookworms, 807–1221 million infected with *Ascaris*, and 604–795 million infected with *Trichuris* [3]. Extensive and detrimental impacts of HAT infections have been reported on human growth, nutrition, fitness, stature, metabolism, cognition, immunity, school attendance/performance, earnings, and pregnancy [3],[4],[5],[6]. A recent and thorough meta-analysis of deworming studies in children demonstrated that deworming children in areas for which HAT parasites are prevalent results in statistically significant improvements in almost all primary outcome measures (weight, height, mid-upper arm circumference, and triceps skin fold) and in all secondary outcome measures (*e.g.*, weight-for-age, height-for-age, …) [5].

Although HAT infections are one of the most prevalent and important infectious diseases in the world, few treatment options exist. The World Health Organization (WHO) has approved two classes of compounds (anthelmintics) for treatment of intestinal nematode parasites: the benzimidazoles (*i.e.*, mebendazole and albendazole) and the nicotinic acetylcholine receptor (nAChR) agonists (*i.e.*, levamisole and pyrantel) [7]. For practical reasons (*e.g.*, efficacy against hookworm, single dose application, weight-independent dosing), only one drug, albendazole, is the drug of choice for Mass Drug Administration [7],[8]. Given the limited number of drugs available, the enormous numbers of people to be treated, and the necessity for repeated treatment due to high reinfection rates and population dynamics of the parasites, the emergence of resistance to existing anthelmintics (already an enormous problem for veterinary anthelmintics [9]) poses a serious threat to large-scale deworming efforts. Thus there have been urgent and repeated calls for the development of new human anthelmintics [6],[7],[10].

In the past 30 years, only one new anthelmintic to treat human HAT infections has reached the clinic, tribendimidine. Tribendimidine, a symmetrical diamidine derivative of amidantel, is a broad-spectrum anthelmintic drug developed by the Chinese National Institute of Parasitic Diseases during the 1980s [11]. It was approved for human use by the China State Food and Drug Administration in 2004 and is currently undergoing clinical testing in China [11],[12]. Laboratory and clinical investigations demonstrate that this drug is safe and has a broad spectrum of single-dose activity against parasitic nematode infections in humans, including against *Ascaris*, hookworms and *Strongyloides stercoralis* with reported cure rates of 92–96%, 52–90%, and 55% respectively [11],[12]. A phase IV clinical trial of tribendimidine recently has been conducted in China [13]. In addition to intestinal nematode infections, tribendimidine has also shown *in vivo* efficacy against trematodes and tapeworms [12],[14]. Tribendimidine is an important new drug with broad anti-parasite activity.

Although tribendimidine is a promising new anthelmintic, virtually nothing is known about its mechanism of action, about whether or not nematodes can develop resistance to it, and, if so, about the molecular mechanism(s) associated with resistance. Such information is vital for understanding whether tribendimidine represents a new type of anthelmintic, for predicting how resistance might develop, for monitoring resistance in the field, and for determining how to rotate/combine it with other anthelmintics. Although the required mechanistic and resistance studies are difficult to conduct with parasitic nematodes, they can readily be carried out using the laboratory nematode, *Caenorhabditis elegans*. *C. elegans* has a rapid life cycle, is susceptible to most known anthelmintics, and is amenable to mutagenesis, large-scale forward genetic screens, genetics, and relatively quick gene mapping and cloning. As such, *C. elegans* has been used to discover and/or clarify the mechanisms of action and resistance of almost all known anthelmintics [15]. Here we demonstrate that *C. elegans* is susceptible to tribendimidine and that *C. elegans* mutants resistant to tribendimidine can readily be isolated. Detailed studies of tribendimidine-resistant and other anthelmintic resistant mutants demonstrate that tribendimidine unambiguously is a member of the nAChR class of anthelmintics of the same subtype as levamisole and pyrantel.

## Materials and Methods

### *C. elegans* strains

*C. elegans* strains were cultured using standard techniques including the use of *Escherichia coli* strain OP50 as standard food source [16]. The following strains were used for tribendimidine resistant mutants (*trb*) outcrossing, chromosome mapping, and complementation testing: Bristol N2, *dpy-5(e61)*, *dpy-11(e224)*, and Hawaiian mapping strain CB4856. The following levamisole-resistant mutant alleles were used: *lev-1 (e221)*, *unc-29 (e293)*, *unc-38(e264)*, *unc-74 (e883)*, *unc-63(x13)*, *lev-8(x15)*, *lev-9(x16)*, *unc-50(e306)*, *unc-22 (e66)*, *unc-22(s12)*, *lev-10(x17)*, *lev-11(x12)*. In addition, the aldicarb resistant mutant *unc-10(e102)* and the levamisole-insensitive nicotinic acetylcholine receptor mutant *acr-16(ok789)* were also used. The strain PD4793 is a strain of *C. elegans* with various green fluorescent protein (GFP) markers integrated on chromosome V.

### Reagents

Tribendimidine was provided by the National Institute of Parasitic Diseases and Chinese Center for Disease Control and Prevention (Shanghai, China). Levamisole and pyrantel were prepared from powder from Acros (cat. no. 187870100) and Sigma (P7674), respectively. A stock solution of tribendimidine at 4 mg/mL was prepared in 1% DMSO in sterile distilled water for all assays. For all plate and well assays, the final concentration of DMSO was ≤0.1%, which both others and we have found has no detectable effect on *C. elegans*, health, movement or development ([17], Y.H. and R.V.A., unpublished data). Levamisole and pyrantel were freshly dissolved in sterile distilled water. The chemical structures of all three drugs, tribendimidine, levamisole, and pyrantel, are shown in Figure S1. The recipe for NG and ENG plates can be found in [18]. Special S medium (sS medium) is a modification of standard S medium used for *C. elegans* liquid culturing [19] in which the pH has been raised to 7.3 and CaCl_2_ has been omitted (we found that tribendimidine is mostly inactivated at pH 6.0, the pH of regular S medium; furthermore CaCl_2_ precipitates at pH 7.3, hence the requirement that it be omitted). We have quantitatively confirmed that *C. elegans* health, development, movement, and brood sized are not affected by using sS medium in place of S medium.

### Genetic screening for resistance mutants, complementation testing, gene mapping, and molecular characterizations

A large population of synchronized 4^th^ larval stage (L_4_) worms was mutagenized in a 30 mM ethyl methanesulfonate (EMS) as per standard protocol [19]. The mutagenized P_0_ animals were grown on OP50-seeded ENG plates at 20° overnight until gravid adults. F_1_ embryos were isolated from these adults using standard bleaching protocols [18]. After hatching overnight at 25° in M9 medium [19], the F_1_ L_1_ larvae were plated and grown on OP50-seeded ENG plates at 20° for 3 days until gravid adults. These adults were bleached to produce F_2_ embryos and then hatched overnight in M9 to produce F_2_ L_1_ larvae. These F_2_ L_1_ larvae were plated onto ENG plates and grown until the L_4_ stage at 20°, at which point they were washed off the plates, rinsed in sS medium, and then pipetted into 48-well plates at a density of 20–30 worms/well along with 60 µg/mL tribendimidine, 20 µL OP50 (OD_600_ = 3.0 in sS medium), and sS medium up to 200 µL final volume. Tribendimidine-exposed worms were then incubated at 15° overnight. Any nematodes that were motile (*i.e.*, resistant to tribendimidine-induced paralysis) were then transferred out of the wells and grown on NG plates (minus drug) to produce progeny. Progeny from these putative candidates were then placed onto NG plates in which tribendimidine (from the 4 mg/mL stock; see above) was added to a final concentration of 100 µg/mL just prior to pouring of the plates. Of 15 putative candidates identified initially, ten were reconfirmed on these tribendimidine plates. To ensure independence of mutants isolated, we screened only 7,600 F_2_ animals out of a total F_2_ population of 152,000 (which came from a population of mutagenized 25,300 F_1_) for an estimated 7,600 mutagenized F_1_ genomes screened.

The tribendimidine resistant mutants were outcrossed as follows: *trb-1(ye492)* was outcrossed six times using a combination of wild-type N2, *dpy-5(e61)*, and *dpy-11(e224)*; *trb-2(ye493)* was outcrossed six times using a combination of N2 and *dpy-5(e61)*; *trb-3(ye494)* was outcrossed six times using a combination of N2 and *dpy-11(e224)*; and *trb-4(ye494)* was outcrossed three times using N2. In addition, the unlinked double mutants *trb-1(ye492);dpy-11(e224)*, *dpy-5(e61);trb-2(ye493)*, and *trb-3(e494);dpy-11(e224)* were obtained. To do complementation tests among *trb* mutants, homozygous or heterozygous males from outcrossed strains were obtained and these were mated into *trb;dpy* double mutant animals or *trb-4(ye495)* animals that on their own are uncoordinated (Unc). More than 10 cross-progeny (non-Dpy or non-Unc animals) from each cross were placed onto 100 µg/mL tribendimidine toxin plates at 25° for 24 hrs and scored for either 100% or 50% resistance, depending upon whether homozygous or heterozygous males were used. To test for complementation between *trb* mutants and levamisole resistant mutants, we crossed homozygous PD4793 GFP males into each of the following levamisole resistance mutants: *lev-1(e211)*, *lev-8(x15)*, *lev-9(x16)*, *lev-10(x17)*, *lev-11(x12)*, *unc-29(e293)*, *unc-38(e264)*, *unc-50(e306)*, *unc-63(x13)*, *unc-74(e883*), *unc-22(e66)*, and *unc-22(s12)*. Heterozygous males were then crossed into *trb-1(ye492)*, *trb-2(ye493)*, *trb-3(ye494)*, or *trb-4(ye495)* animals. For each of these crosses, 20 GFP cross-progeny were each plated onto either 1 mM levamisole or 100 µg/mL tribendimidine plates (levamisole plates were prepared using a 100 mM stock of levamisole in sterile distilled water). The matching of levamisole and *trb* genes was determined by resistance of half of the cross-progeny on both tribendimidine and levamisole plates. Unambiguous results were obtained as described in the text.

For gene mapping, each *trb* mutant was mapped to specific chromosomes and subregions using CB4856 and single-nucleotide polymorphisms [20]. *trb-1* was mapped near the middle arm of chromosome I, *trb-2* was mapped to the middle region of chromosome X, *trb-3* was mapped to the left arm of chromosome III, and *trb-4* was mapped to the middle region of chromosome IV.

For detecting molecular changes of *trb* alleles in specific levamisole resistance genes, we used the polymerase chain reaction (PCR) to amplify DNA or cDNA isolated from various *trb* mutant animals with the coding region of specific levamisole resistance genes (Table 1). Pfu Ultra HS HF DNA Polymerase from Stratagene (USA) was used for these amplifications. All the sequence results were confirmed with three independent PCR reactions and double-stranded sequencing. Since the *unc-22* gene is very large, we did not sequence in this case. Instead, we did the complementation tests between two different *unc-22* alleles (*e66 and s12*) and all three *trb-4* alleles (*ye495*, *ye496 and ye497*).

**Table 1:** Templates, primers, and target genes for identification of molecular changes associated with *trb-1*, *trb-2*, and *trb-3* alleles.

PCR Template	Primers	Amplified Gene
*trb-1(ye492)* genomic DNA	Upstream primer: 5′-GTTAATGGGACCAAATGACCACGGTTTTG-3′	*unc-63*
	Downstream primer: 5′-CTAAGCAAGAGCCGGCGTGTTATCG-3′	
*trb-2 (ye494)* cDNA	Upstream primer: 5′-CTTATGTGGATACCACAACGG-3′	*lev-8*
	Downstream primer: 5′-TCAGGTGTTAAGAACGTTGATG-3′	
*trb-3 (ye494)* cDNA	Upstream primer: 5′-GTCATGAGTTCACAGCCGCGAGG-3′	*unc-50*
	Downstream primer: 5′- TTAAAGACCGCCGTGTTGGG-3′	

### Intoxication assays

To examine gut morphology, individual L_4_ hermaphrodites were individually picked using an eyelash into wells as described above for resistance screening except tribendimidine was used at 100 µg/mL. The animals were incubated for 24 hours at 25°, pipetted onto an agarose pad with 3 mM sodium azide as an anesthetic, visualized with 600× Nomarski optics on an Olympus IX70 microscope with a 60× PlanApo lens (1.4 NA), and photographed with a cool SNAP HQ^2^ camera (PhotoMetrics, Inc, USA).

For measuring dose-dependent developmental inhibition, we pipetted into the wells of a 48-well plate approximately 20 L_1_ nematodes, 20 µL OP50 (OD_600_ = 3.0), 20 µL drug, and a total volume of 200 µL (sS medium is used as the dilutant for all reagents). Each well contained a specific dose of drug and that dose was repeated for a total of three times per experiment. The microtiter plate was then wrapped in damp paper towels, placed inside a covered plastic box, and incubated at 20° for 60 h. The number of nematodes that did/did not reach gravid adulthood (harboring one or more eggs in their uterus) were tallied for each well. The experiment was independently repeated three times.

A mortality assay was used to determine dose-dependent mortality of nematodes exposed to drugs for 6 days at 25°. From these data the LC_50_, the concentration at which 50% of the nematodes are dead, was calculated. Death was defined as worms that failed to respond to touch, were very pale, and had lost most internal structures. The LC_50_ assay with ∼20 L_4_ animals per well in sS medium was set up as previously described [18], with the exception that different strains were allowed to grow for different amounts of time at 20° from the L_1_ to L_4_ stage prior to testing on drugs in order to reflect slight differences in their growth rates relative to N2 wild type: *trb-4(ye495)*, *lev-1(e211)*, *lev-11(x12)*, and *unc-22(e66)* mutant nematodes were allowed to develop for 48 hr and *trb-1(ye492)*, *trb-2(ye493)*, *trb-3(ye494)*, *lev-8(x15)*, *lev-9(x16)*, *lev-10(x17)*, *unc-29(e293)*, *unc-38(e264)*, *unc-50(e306)*, *unc-63(x13)*, and *unc-74(e883*) were allowed to develop for 45 hours (N2 wild-type animals were used at 44 hours as previously described).

To calculate 64 h brood sizes, individual L_4_ worms were picked up with an eyelash and placed in sS medium in a 48-well plate containing 40 µL OP50 (OD_600_ = 3.0) and a specific dose of tribendimidine. The total volume in each well was 200 µL. Each drug concentration was repeated in five wells per experiment. The plates were incubated for 64 h at 25°. The progeny were then transferred out of the well with a pipette onto an empty NG agar plate for counting. For complete brood sizes of various strains in the absence of drug, individual L_4_ wild-type or *trb* hermaphrodites were picked onto individual OP50-seeded NG plates. Every two days, each adult hermaphrodite was shifted to a new NG plate until it stopped producing offspring. The progeny from the old plates were counted the next day.

### Statistical analyses

LC_50_ values and associated 95% confidence intervals were calculated using the PROBIT algorithm (from XLSTAT add-on to EXCEL). Dose-response curves were plotted using Prism 5 (GraphPad Software Inc., La Jolla, CA). For brood size data, statistical analyses were carried out using Prism 5, as were pair-wise comparisons between groups via one-way analysis of variance (ANOVA) and Tukey's HSD test.

## Results

### Wild-type *C. elegans* is susceptible to tribendimidine

**Figure 1:**
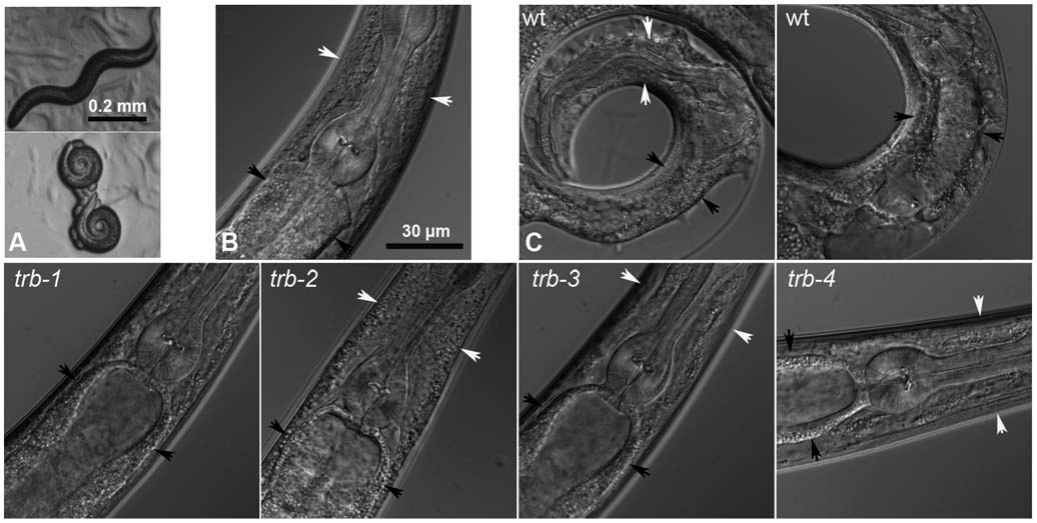
Intoxication of *C. elegans* by tribendimidine. A. L_4_ worms exposed to no drug (upper) or 100 µg/mL tribendimidine (lower) for 24 h at 25° and photographed at 30× magnification. Tribendimidine causes most *C. elegans* animals to coil. Scale bar applies to both panels. B and C. 600× magnification of animals under various conditions. B. Wild-type control animal without drug showing healthy intestine (between black arrowheads). White arrowheads (here and in other panels) point to cuticular regions within which the pharyngeal isthmus is contained. C. Animals on 100 µg/mL tribendimidine. Top row: wild-type animals on tribendimidine. Significant damage to the intestine (between black arrowheads) is evident, as well as degradation of the body around the pharyngeal isthmus of the left-most animal. Bottom row: tribendimidine resistant animals on tribendimidine. Note, all have healthy intestines and no degradation of body cavity is evident. Scale bar in B applies to all images in B and C. wt = wild type. Alleles used are *trb-1(ye492)*, *trb-2(ye493)*, *trb-3(ye494)*, and *trb-4(ye495)*.

Since there were no previous reports of the effects of tribendimidine on *C. elegans*, we incorporated the drug into standard nematode growth plates at 100 µg/mL and exposed the nematode to the drug at 25° for 24 hour. Under these conditions, the nematodes become paralyzed, although they are all still alive based on their coloration and the fact that they continue to lay eggs. The vast majority of these animals are coiled up and contracted (Figure 1A); a few are contracted but not coiled up. When placed in liquid media at the same concentration, wild-type *C. elegans* rapidly become straightened; only the extreme ends of the animal are able to move. After 24 h exposure to drug, most of the animals become coiled and immobile as on plates, although they are still alive since they lay eggs and will respond to direct touch or vigorous shaking of the microtiter plate. When these animals are mounted for observation at higher magnification, their internal morphology has degenerated, and damage to multiple tissues is evident, including shrinkage of the intestine away from the body wall (Figure 1B, C). The neuromuscular system is probably also damaged based on the motility defects described above.

**Figure 2:**
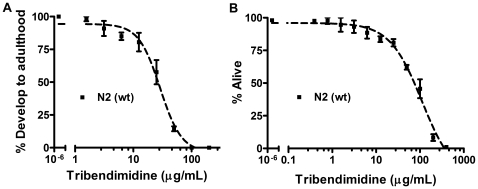
Dose response of wild-type *C. elegans* to tribendimidine. A. Response of wild-type (N2) *C. elegans* to tribendimidine as measured by the effect of various doses of the drug on the ability of larvae to develop to adulthood. B. Response of *C. elegans* to tribendimidine measured by the effect of various doses of the drug on viability. LC_50_ value is given in Table 2. For both A and B, each data point represents on average 180 nematodes (n = 3 repeats; 3 replicate wells per repeat). Error bars represent standard error of the mean for the three independent experiments. For converting to a mM dose, 100 µg/mL tribendimidine is equivalent to 0.22 mM.

To quantify the effects of tribendimidine on *C. elegans*, we performed a number of quantitative assays. First, we examined the response of *C. elegans* to tribendimidine based on what percentage of L_1_ larvae are able to develop to the gravid adult stage at varying doses of the drug (Figure 2A). We find that *C. elegans* demonstrates a well-behaved, dose-dependent response to tribendimidine with regards to inhibition of larval development (Figure 2A), with an IC_50_ (inhibitory concentration at which 50% of the larvae are unable to complete development at these conditions) of 18.4 µg/mL (95% confidence interval 16.2–22.3 µg/mL).

Next, we placed *C. elegans* L4 animals in wells at varying concentrations of the drug and assayed for mortality after 6 days at 25°. We find that *C. elegans* demonstrates a well-behaved, dose-dependent response to tribendimidine with respect to mortality (Figure 2B). The LC_50_ value (concentration at which half the animals are dead) is 54.4 µg/mL (Table 2). As discussed below, we also found that tribendimidine is able to produce a dose-dependent decrease in *C. elegans* progeny production.

**Table 2:** LC_50_ values associated with experimental results.

Figure number	Genotype	Drug	LC_50_ (µg/mL)	95% Confidence Interval
2B	N2 (wt)	Tribendimidine	54.4	45.2–63.5
3	N2 (wt)	Tribendimidine	50.0	45.2–72.2
6	N2 (wt)	Levamisole	26.8	23.0–31.2
	*trb-1(ye492)*		363.1	340.6–387.2
	*trb-2(ye493)*		437.5	Very wide
	*trb-3(ye494)*		412.2	361.7–469.7
	*trb-4(ye495)*		217.1	176.2—267.6

### Isolation of *C. elegans* tribendimidine-resistant mutants

A forward genetic screen was carried out to find *C. elegans* mutants resistant to tribendimidine (see Materials and Methods for details). After screening 7,600 mutagenized F_2_ animals, a total of ten resistant animals were identified that bred true in subsequent generations. Initial identification and confirmation of resistance were based on the fact that all were motile and healthy at concentrations of tribendimidine that paralyze and intoxicate wild type. Complementation testing among these ten different alleles revealed they fell into four groups that we called *trb-1* (five alleles), *trb-2* (1 allele), *trb-3* (1 allele), and *trb-4* (3 alleles) (*trb* for **tr**i**b**endimidine resistant). All *trb* mutants are clearly resistant to tribendimidine intoxication. In contrast to wild-type animals, *trb* animals exposed to tribendimidine display a healthy body morphology (Figure 1C) similar to that of wild-type animals unexposed to the anthelmintic (Figure 1B).

**Figure 3:**
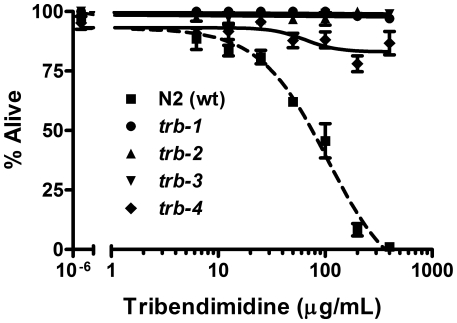
*trb* mutants resist tribendimidine-induced mortality. The response of wild-type N2 and four *trb* mutant animals to various doses of tribendimidine as measured by viability after 6 days at 25°. Each data point represents on average 180 worms (n = 3 repeats; 3 replicate wells per repeat). Error bars represent standard error of the mean for the three independent experiments. Allele designations are as in Figure 1. The LC_50_ value for wild-type is reported in Table 2.

To quantitatively demonstrate resistance, we measured the ability of wild-type (N2) animals and animals from one representative allele of each complementation group— namely *trb-1(ye492)*, *trb-2(ye493)*, *trb-3(ye494)*, and *trb4 (ye495)*—to survive over a wide dose range of tribendimidine (Figure 3). At tribendimidine concentrations where most or all of the wild-type nematodes are dead (*e.g.*, ≥200 µg/mL), the *trb-*mutant nematodes are mostly or all alive. As opposed to wild-type animals, we did not calculate an LC_50_ value for any of the *trb* mutants since there was no concentration in this experiment at which ≥50% of any *trb* mutant nematodes died. Larvae from all four *trb* mutants are also resistant to intoxication since they mature to adults at doses that inhibit wild-type larval development (unpublished observation).

**Figure 4:**
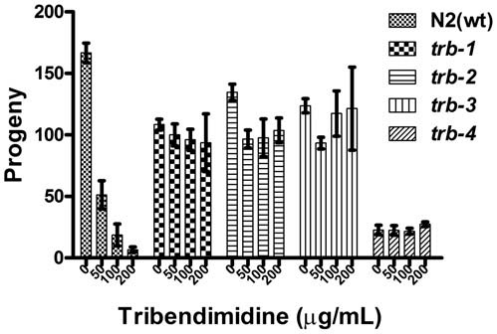
*trb* mutants resist tribendimidine-induced sterility. Brood sizes of N2 wild-type (wt) and *trb* mutant hermaphrodites after 64 h on either no drug or three different doses of tribendimidine. The experiment was done at 25° and repeated a total of three times with five wells/experiment/genotype/dose (thus each bar represents brood sizes from 15 worms). Whereas the brood size of wild-type animals decreases upon exposure to tribendimidine (P<0.01 for 0 vs. 50 µg/mL; P<0.001 for 0 vs. 100 and 200 µg/mL), the brood sizes of all four *trb* mutants are unaffected even by the highest dose of tribendimidine tested (P>0.05 for all pair-wise comparisons of all doses for any given mutant). The relative brood sizes of wild-type animals at 50, 100, and 200 µg/mL are respectively 31%, 12%, and 5% of the brood size without toxin. The brood size of *trb-4* animals in the absence of drug is clearly lower than that of the other genotypes. Further data and discussion of brood sizes of wild-type vs. *trb* animals in the absence of drug is given in Figure S2. Error bars represent standard deviations. Allele designations are as in Figure 1.

Resistance to tribendimidine was also confirmed using a quantitative brood size assay [21],[22] for all four *trb* mutants. Wild-type *C. elegans* hermaphrodites show a dose-dependent decrease in brood size production upon exposure to tribendimidine (Figure 4). In contrast, all *trb* mutant hermaphrodites exposed to even high doses of tribendimidine show healthy brood sizes that are statistically the same as brood sizes in the absence of the anthelmintic, confirming their resistance (Figure 4).

### *trb* mutant animals are resistant to levamisole and pyrantel

**Figure 5:**
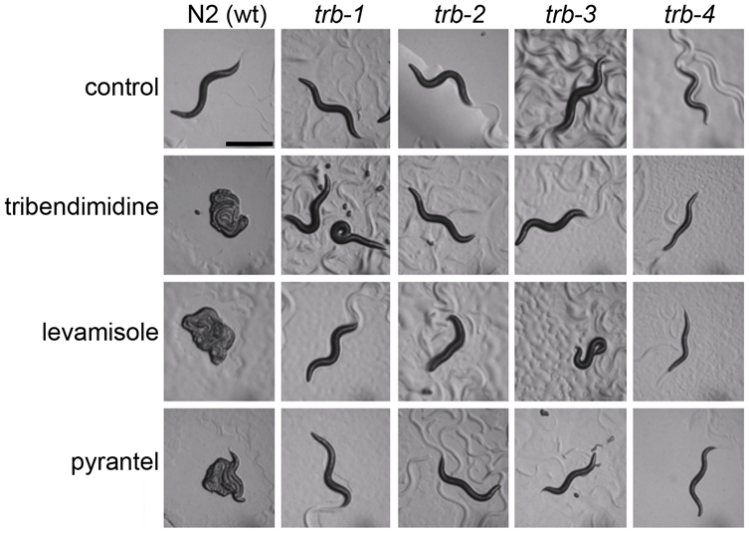
*trb* mutants are qualitatively resistant to levamisole, and pyrantel. L_4_ hermaphrodites of the genotype indicated above the panels were seeded onto plates with no anthelmintic (control), 0.22 mM tribendimidine (100 µg/mL), 1 mM levamisole, or 2 mM pyrantel. Animals were incubated at 25° for 24 h. Photos were taken under the dissecting scope at 30× magnification (scale bar in first panel = 0.5 mm). Wild-type (wt) N2 animals are immotile and cluster on plates with any of these drugs. *trb-1*, *trb-2*, and *trb-3* animals are motile on all three drugs and do not cluster, indicating their resistance to all three. *trb-4* animals are motile on these drugs but are Unc even in the absence of drugs and therefore cannot move normally. Some of these animals cluster and some do not on the drugs, but they are clearly resistant. Allele designations are as in Figure 1.

In the course of our studies, we noticed that tribendimidine stimulated egg-laying in wild-type animals, a behavior that had been previously reported for wild-type *C. elegans* exposed to the nAChR agonist anthelmintic levamisole [23]. We therefore speculated that tribendimidine might have a similar mechanism of action as levamisole. If so, then one might hypothesize that *trb* resistant animals might have altered responses to levamisole. To test this hypothesis, we place *trb* mutant animals on levamisole-containing plates. Whereas wild-type animals become paralyzed and aggregate when exposed to levamisole for 24 h, *trb* mutant animals are motile and mostly fail to aggregate on levamisole (Figure 5). Identical results were obtained with pyrantel, another nAChR agonist anthelmintic of the same subtype and mechanism of action as levamisole (Figure 5; pyrantel and levamisole are collectively known as the L-subtype nAChR agonists [24]).

**Figure 6:**
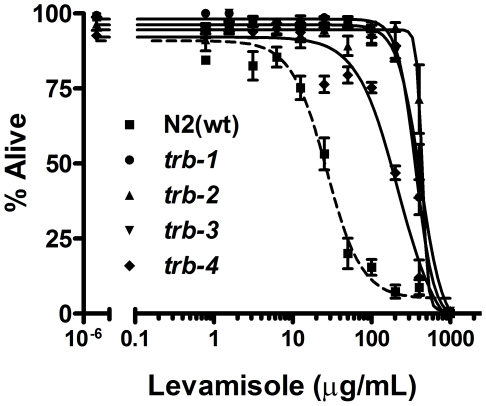
*trb* mutants are quantitatively resistant to levamisole. Mortality of wild-type (wt) N2 and *trb-1*, *-2*, *-3*, and *-4* mutant animals exposed to varying doses of levamisole for 6 days at 25°. Each data point represents on average 180 worms (n = 3 repeats; 3 replicate wells per repeat). Error bars represent standard error of the mean for the three independent experiments. Allele designations are as in Figure 1. The LC_50_ value for each genotype is reported in Table 2. For converting to a mM dose, 100 µg/mL levamisole is equivalent to 0.42 mM.

These data indicate that tribendimidine-resistant *C. elegans* are also resistant to L-subtype nAChR agonists. To quantitatively confirm this result, we performed dose-dependent mortality assays of *trb-1(ye492)*, *trb-2(ye493)*, *trb-3(ye494)* and *trb-4(ye495)* hermaphrodites on levamisole (Figure 6). Resistance can be readily discerned at specific concentrations of levamisole; for example at 100 µg/mL only 20% of wild-type animals are alive whereas 99.5%, 95%, 99% and 81.5% of *trb-1(ye492)*, *trb-2(ye493)*, *trb-3(ye494)* and *trb-4(ye495)* animals are alive (P = 0.001, ANOVA Tukey's test). Based on LC_50_ values (Table 2), these mutants are 8–16 fold more resistant than wild-type animals to levamisole. We also performed dose-dependent mortality assays of wild-type, *trb-2(ye493)*, and *trb-3(ye494)* animals on pyrantel (Figure S3). Although pyrantel is not as effective as levamisole at killing *C. elegans* ([16]; this study), animals from both *trb* mutants are resistant to pyrantel relative to wild-type animals.

**Figure 7:**
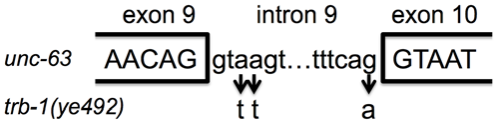
Mutations in *trb-1(ye492)* are associated with exon/intron boundaries of the *unc-63* gene. Above, nucleotide sequence of the *unc-63* gene at the exon 9/intron 9 and intron 9/exon 10 boundaries. Below, three altered nucleotides in *trb-1(ye492)* as indicated by down arrows. Assuming the mutant intron is not spliced, then inclusion of intron 9 would result in a translated protein with two missense mutations at amino acids 458 and 459 followed by a premature stop codon (the full length protein is normally 502 aa).

Extensive screens for *C. elegans* resistant to levamisole have been carried out and have identified a number of genes that mutate to levamisole resistance [25]. Since mutations in *trb-1*, *-2*, *-3*, and *-4* resist levamisole, we hypothesized that these mutations might exist in genes known to mutate to levamisole resistance. We mapped the *trb*-1, -2, -3, and -4 genes to various segments of chromosomes I, X, III, and IV, respectively (see Materials and Methods for details). Each *trb* mutant was then subjected to genetic complementation tests against known levamisole-resistant mutants located on the same chromosome, to wit *trb-1* was tested against *unc-29*, *unc-38*, *unc-74*, and *unc-63*, (but not *lev-11* or *lev-10* mutants since these were far away on the right arm of chromosome I); *trb-2* was tested against *lev-8* and *lev-9* mutants; *trb-3* was tested against the *unc-50* mutant; and *trb-4* was tested against two alleles of the *unc-22* mutant and the *lev-1* mutant (alleles given in Materials and Methods). We found that *trb-1(ye492)*,*trb-2(ye493)*, *trb-3(ye494)*, and *trb-4(ye495)* each unambiguously failed to complement just one mutant, namely *unc-63(x13)*, *lev-8(x15)*, *unc-50(e306)* and *unc-22(e66 or s12)* respectively. To confirm these identities, we sequenced genomic DNA or cDNA isolated from *trb-1(ye492)*, *trb-2(ye493)*, and *trb-3(ye494)* animals (*trb-4/unc-22* is an extremely large locus covering more than 37 kb of DNA and hence was left out of sequencing analyses). For *trb-2(ye493)* and *trb-3(ye494)*, we found that these alleles are associated with point mutations in *lev-8* (tryptophan 164 to a stop codon) and *unc-50* (serine 261 to leucine) respectively. The mutation in *trb-2(ye493)* is predicted to result in truncation of the C-terminal 70% of the LEV-8 protein, consistent with a null mutant. *trb-3(ye494)* is associated with a non-conservative change in an amino acid that is also conserved in *unc-50* homologues of other nematodes such as *Caenorhabditis briggsae* and *Brugia malayi*, consistent with the fact it might reduce or eliminate function. For *trb-1(ye492)*, we found three alterations in nucleotides located in intron 9 of the *unc-63* gene (Figure 7). These alterations occur in conserved intron sequences and can be required for normal splicing [26],[27]. Thus, the resistance, mapping, complementation, and sequence data indicate that the four complementation groups identified for tribendimidine resistance all occur in genes previously found in screens for levamisole resistance.

### Levamisole-resistant mutants are resistant to tribendimidine

**Figure 8:**
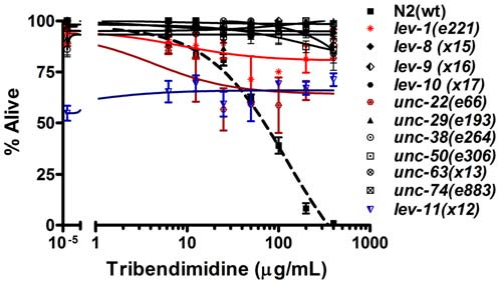
Levamisole-resistant mutants are resistant to tribendimidine. Eleven mutants isolated based on their ability to resist levamisole were subjected to dose-dependent tribendimidine mortality assays. Each data point represents viability for three independent trials for each mutant, with three wells per trial (average of 180 worms per data point). Error bars represent standard error of the mean.

To determine how much overlap there is between genes that mutant to levamisole resistance and tribendimidine resistance, we took *C. elegans* strains mutated for eleven levamisole-resistance genes and performed dose-dependent tribendimidine mortality assays (Figure 8). Taking into account of that some of these mutants (*i.e.*, *unc-22* and *lev-11*) have compromised health even in the absence of drug, these data clearly show that all eleven mutants are resistant to tribendimidine as demonstrated by their robust survival at doses of the drug that are highly lethal to wild-type (≥200 µg/mL; Figures 8 and S4). Thus, for eleven out of eleven levamisole resistant mutants tested, they are also resistant to tribendimidine.

## Discussion

The free living nematode *C. elegans* has been extensively used in the study of anthelmintics [15],[25],[28]. *C. elegans* is considered an excellent model for anthelmintic mode of action and resistance and has proven invaluable in finding the mechanism of action of almost all anthelmintics in use today. There are many excellent examples of forward genetic screens to discover mutants that allow *C. elegans* to resist anthelmintics, thereby leading to an understanding of their mechanism of action and mechanisms whereby resistance can develop, including screens for resistance to levamisole [29], benzimidazoles [17], aldicarb [30], ivermectin [31],[32], and most recently amino-acetylnitriles [33].

Using the same approach, we have demonstrated that new anthelmintic tribendimidine is an L-subtype nAChR agonist of the same family as levamisole and pyrantel. Tribendimidine causes changes in the egg-laying behavior of *C. elegans* grossly similar to levamisole. More importantly, a forward genetic screen for *C. elegans* animals resistant to tribendimidine resulted in the isolation and identification of four mutants that are also resistant to both levamisole and pyrantel and that in fact mutate the same genes that give rise to levamisole resistance. Furthermore, a retrospective study of eleven mutant strains isolated based on their resistance to levamisole demonstrated that all of these mutants are also resistant to tribendimidine. In contrast to these levamisole-resistant mutants, we find that two mutants that affect signaling at the neuromuscular junction independent of levamisole, namely *acr-16(ok789)* animals, which lack a levamisole-insensitive nACh receptor [34], and *unc-10(e102)* animals, which are resistant to the cholinesterase inhibitor aldicarb [30], are qualitatively sensitive to tribendimidine (Figure S5). Consistent with the fact that tribendimidine does not behave like an cholinesterase inhibitor we find that tribendimidine at 0.5 mM, like levamisole at 1 mM, paralyzes animals in seconds, most noticeably at the tip of head, versus cholinesterase inhibitors that take many minutes to affect wild type and contract the body before the head [29].

Thus, although not necessarily intuitive based on its chemical structure (Figure S1), tribendimidine intoxicates *C. elegans* using the same pathway as levamisole and thus shares the same mechanism of action as the L-subtype nAChR agonists levamisole and pyrantel. Given the extensive and complete correspondence in the nematode *C. elegans* between levamisole resistance and tribendimidine resistance, we are certain that tribendimidine will have the same mechanisms of action and resistance as levamisole/pyrantel in parasitic nematodes as well.

There are several practical applications of these results. For treating hookworm infections, the intestinal parasitic nematode with the highest disease burden , the benzimidazole albendazole is currently the treatment of choice since it has much better cure rates than levamisole and pyrantel as a single dose and can be given as a fixed dose, unlike the nAChR agonists that are given as dose/weight [7],[35]. Recent work with tribendimidine suggests that it is superior to levamisole or pyrantel at a single dose and comparable to single-dose albendazole in treating *Ascaris* or hookworms [11],[12]. Our data indicate that in places where resistance to benzimidazoles exists or is suspected (*e.g.*, in Mali, Zanzibar, Vietnam [10],[36],[37]), tribendimidine would be a good alternative since its mechanism of action is different from that of the benzimidazoles. However, tribendimidine would be a poor choice in places where nAChR agonist resistance exists or is suspected (*e.g.*, in Australia [37]). Furthermore our data indicate that tribendimidine would be useful in combinatorial anthelmintic strategies, such as with benzimidazoles [36], but not in others, such as with levamisole or pyrantel since it shares the same mechanism of action. Our data also highlight the importance of determining the molecular changes associated with L-subtype nAChR agonist resistance in human parasitic nematodes since these changes would allow us to simultaneously track resistance to tribendimidine, levamisole, and pyrantel.

Our study highlights the utility of using *C. elegans* in studying the mechanism of action of anthelmintics used for clinical and veterinary use. This laboratory nematode allows one to rapidly uncover important aspects of new anthelmintic mechanism of action and resistance and can inform how to design strategies for maximizing anthelmintic therapy and minimizing the development of anthelmintic resistance.

## Supporting Information

Figure S1 Structures of all the drugs used in this study. A. tribendimidine. B. levamisole. C. pyrantel.
(0.73 MB TIF)

Figure S2 The total brood sizes of wild-type and tribendimidine resistant animals in the absence of tribendimidine at three different temperatures. Pair-wise comparisons between wild-type (wt) N2 and *trb-1*, *trb-2*, or *trb-3* at each temperature indicate that the total brood sizes are not significantly different at any given temperature (P>0.05). The total brood size of *trb-4* mutant animals at each temperature is different from the corresponding wild-type brood size (P<0.001). Error bars represent standard deviations. n = 5 animals for all bars except n = 4 for N2 and *trb-2* brood sizes at 25°.
(4.40 MB TIF)

Figure S3 *trb-2* and *trb-3* mutant animals are resistant to pyrantel. Standard mortality assays were carried out for wild-type (wt) N2 and *trb-2* and *trb-3* mutant animals as described in the main text. The data come from three replicate experiments with an average of 180 animals per data point. * = P value relative to N2<0.05; ** = P value relative to N2<0.01; *** = P value relative to N2<0.001 (ANOVA analysis, Tukey's HSD test). Error bars represent standard error of the mean.
(0.11 MB TIF)

Figure S4 Quantitative resistance of levamisole-resistant mutants at 200 µg/mL tribendimidine. Data are taken from the 200 µg/mL dose in Figure 8.
(0.18 MB TIF)

Figure S5 Semi-quantitative analysis of various mutants on tribendimidine (Tri). L4 staged animals of the indicated genotype were placed in wells with the indicated amount of tribendimidine and incubated for 24 h at 25°. The *acr-16* and *unc-10* mutant animals are clearly susceptible to tribendimidine as shown by the fact that they are as paralyzed as wild-type animals by the drug at all concentrations and that they are more pale in color than wild-type animals even at lower drug concentrations. *trb-1* mutant animals were included as a resistant control. Scale bar is 1 mm.
(4.93 MB TIF)

## References

[1] Hotez PJ (2008). Forgotten people, forgotten diseases : the neglected tropical diseases and their impact on global health and development.

[2] Hotez PJ, Molyneux DH, Fenwick A, Kumaresan J, Sachs SE (2007). Control of neglected tropical diseases.. N Engl J Med.

[3] Bethony J, Brooker S, Albonico M, Geiger SM, Loukas A (2006). Soil-transmitted helminth infections: ascariasis, trichuriasis, and hookworm.. Lancet.

[4] Albonico M, Allen H, Chitsulo L, Engels D, Gabrielli AF (2008). Controlling Soil-Transmitted Helminthiasis in Pre-School-Age Children through Preventive Chemotherapy.. PLoS Negl Trop Dis.

[5] Hall A, Hewitt G, Tuffrey V, de Silva N (2008). A review and meta-analysis of the impact of intestinal worms on child growth and nutrition.. Matern Child Nutr.

[6] Hotez PJ, Brindley PJ, Bethony JM, King CH, Pearce EJ (2008). Helminth infections: the great neglected tropical diseases.. J Clin Invest.

[7] Keiser J, Utzinger J (2008). Efficacy of current drugs against soil-transmitted helminth infections: systematic review and meta-analysis.. JAMA.

[8] Smits HL (2009). Prospects for the control of neglected tropical diseases by mass drug administration.. Expert Rev Anti Infect Ther.

[9] Kaplan RM (2004). Drug resistance in nematodes of veterinary importance: a status report.. Trends Parasitol.

[10] Flohr C, Tuyen LN, Lewis S, Minh TT, Campbell J (2007). Low efficacy of mebendazole against hookworm in Vietnam: two randomized controlled trials.. Am J Trop Med Hyg.

[11] Xiao SH, Hui-Ming W, Tanner M, Utzinger J, Chong W (2005). Tribendimidine: a promising, safe and broad-spectrum anthelmintic agent from China.. Acta Trop.

[12] Steinmann P, Zhou XN, Du ZW, Jiang JY, Xiao SH (2008). Tribendimidine and Albendazole for Treating Soil-Transmitted Helminths, *Strongyloides stercoralis* and *Taenia* spp.: Open-Label Randomized Trial.. PLoS Negl Trop Dis.

[13] Zhang JH, Xiao SH, Wu ZX, Qiu DC, Wang SH (2008). Tribendimidine enteric coated tablet in treatment of 1,292 cases with intestinal nematode infection–a phase IV clinical trial.. Chin J Parasitol Parasit Dis.

[14] Xiao SH, Jian X, Tanner M, Yong-Nian Z, Keiser J (2008). Artemether, artesunate, praziquantel and tribendimidine administered singly at different dosages against *Clonorchis sinensis*: a comparative *in vivo* study.. Acta Trop.

[15] Geary TG, Thompson DP (2001). *Caenorhabditis elegans*: how good a model for veterinary parasites?. Vet Parasitol.

[16] Brenner S (1974). The genetics of *Caenorhabditis elegans*.. Genetics.

[17] Driscoll M, Dean E, Reilly E, Bergholz E, Chalfie M (1989). Genetic and molecular analysis of a *Caenorhabditis elegans* beta-tubulin that conveys benzimidazole sensitivity.. J Cell Biol.

[18] Bischof LJ, Huffman DL, Aroian RV (2006). Assays for toxicity studies in *C. elegans* with Bt crystal proteins.. Methods Mol Biol.

[19] Sulston J, Hodgkin J, Wood WB (1988). Methods.. The nematode Caenorhabditis elegans.

[20] Davis MW, Hammarlund M, Harrach T, Hullett P, Olsen S (2005). Rapid single nucleotide polymorphism mapping in *C. elegans*.. BMC Genomics.

[21] Griffitts JS, Whitacre JL, Stevens DE, Aroian RV (2001). Bt toxin resistance from loss of a putative carbohydrate-modifying enzyme.. Science.

[22] Wei JZ, Hale K, Carta L, Platzer E, Wong C (2003). *Bacillus thuringiensis* crystal proteins that target nematodes.. Proc Natl Acad Sci U S A.

[23] Kim J, Poole DS, Waggoner LE, Kempf A, Ramirez DS (2001). Genes affecting the activity of nicotinic receptors involved in *Caenorhabditis elegans* egg-laying behavior.. Genetics.

[24] Martin RJ, Verma S, Levandoski M, Clark CL, Qian H (2005). Drug resistance and neurotransmitter receptors of nematodes: recent studies on the mode of action of levamisole.. Parasitology.

[25] Jones AK, Buckingham SD, Sattelle DB (2005). Chemistry-to-gene screens in *Caenorhabditis elegans*.. Nat Rev Drug Discov.

[26] Aroian RV, Levy AD, Koga M, Ohshima Y, Kramer JM (1993). Splicing in *Caenorhabditis elegans* does not require an AG at the 3′ splice acceptor site.. Mol Cell Biol.

[27] Mount SM (1982). A catalogue of splice junction sequences.. Nucleic Acids Res.

[28] Holden-Dye L, Walker RJ (2007). Anthelmintic drugs.. WormBook.

[29] Lewis JA, Wu CH, Berg H, Levine JH (1980). The genetics of levamisole resistance in the nematode *Caenorhabditis elegans*.. Genetics.

[30] Nguyen M, Alfonso A, Johnson CD, Rand JB (1995). *Caenorhabditis elegans* mutants resistant to inhibitors of acetylcholinesterase.. Genetics.

[31] Dent JA, Davis MW, Avery L (1997). *avr-15* encodes a chloride channel subunit that mediates inhibitory glutamatergic neurotransmission and ivermectin sensitivity in *Caenorhabditis elegans*.. EMBO J.

[32] Dent JA, Smith MM, Vassilatis DK, Avery L (2000). The genetics of ivermectin resistance in *Caenorhabditis elegans*.. Proc Natl Acad Sci U S A.

[33] Kaminsky R, Ducray P, Jung M, Clover R, Rufener L (2008). A new class of anthelmintics effective against drug-resistant nematodes.. Nature.

[34] Touroutine D, Fox RM, Von Stetina SE, Burdina A, Miller DM (2005). *acr-16* encodes an essential subunit of the levamisole-resistant nicotinic receptor at the *Caenorhabditis elegans* neuromuscular junction.. J Biol Chem.

[35] Utzinger J, Keiser J (2004). Schistosomiasis and soil-transmitted helminthiasis: common drugs for treatment and control.. Expert Opin Pharmacother.

[36] Albonico M, Bickle Q, Ramsan M, Montresor A, Savioli L (2003). Efficacy of mebendazole and levamisole alone or in combination against intestinal nematode infections after repeated targeted mebendazole treatment in Zanzibar.. Bull World Health Organ.

[37] Geerts S, Gryseels B (2001). Anthelmintic resistance in human helminths: a review.. Trop Med Int Health.

