# Supplementary figures and images for: The New Anthelmintic Tribendimidine is an L-type (Levamisole and Pyrantel) Nicotinic Acetylcholine Receptor Agonist
Source: PLoS Negl Trop Dis. 2009 Aug 11;3(8):e499. doi: 10.1371/journal.pntd.0000499 (PMC2715879; doi:10.1371/journal.pntd.0000499)

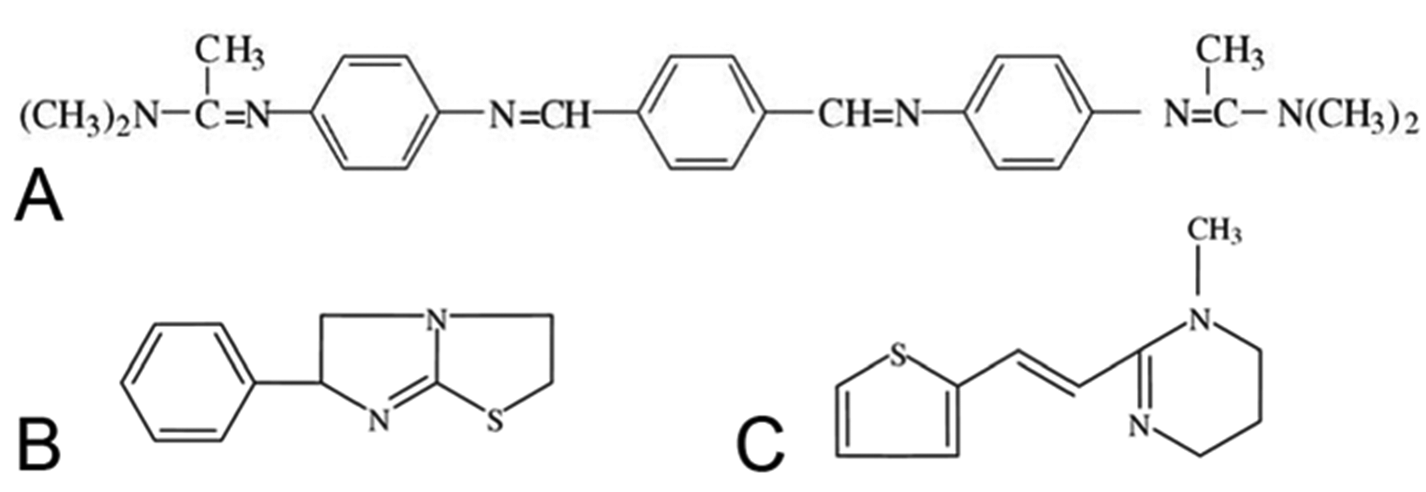

Supplement: Figure S1 — Structures of all the drugs used in this study. A. tribendimidine. B. levamisole. C. pyrantel. (0.73 MB TIF) [file pntd.0000499.s001.tif]

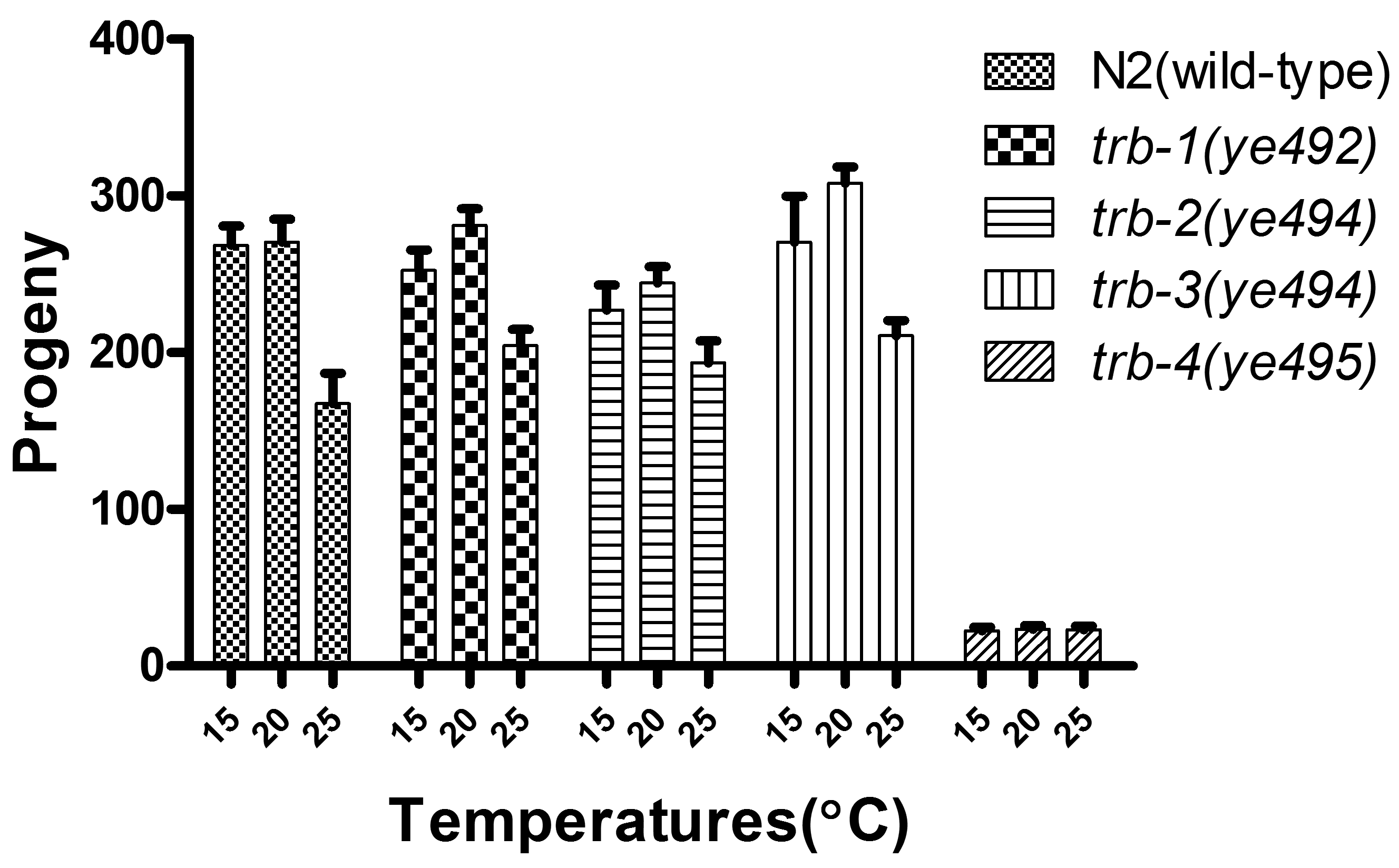

Supplement: Figure S2 — The total brood sizes of wild-type and tribendimidine resistant animals in the absence of tribendimidine at three different temperatures. Pair-wise comparisons between wild-type (wt) N2 and trb-1, trb-2, or trb-3 at each temperature indicate that the total brood sizes are not significantly different at any given temperature (P>0.05). The total brood size of trb-4 mutant animals at each temperature is different from the corresponding wild-type brood size (P<0.001). Error bars represent standard deviations. n = 5 animals for all bars except n = 4 for N2 and trb-2 brood sizes at 25°. (4.40 MB TIF) [file pntd.0000499.s002.tif]

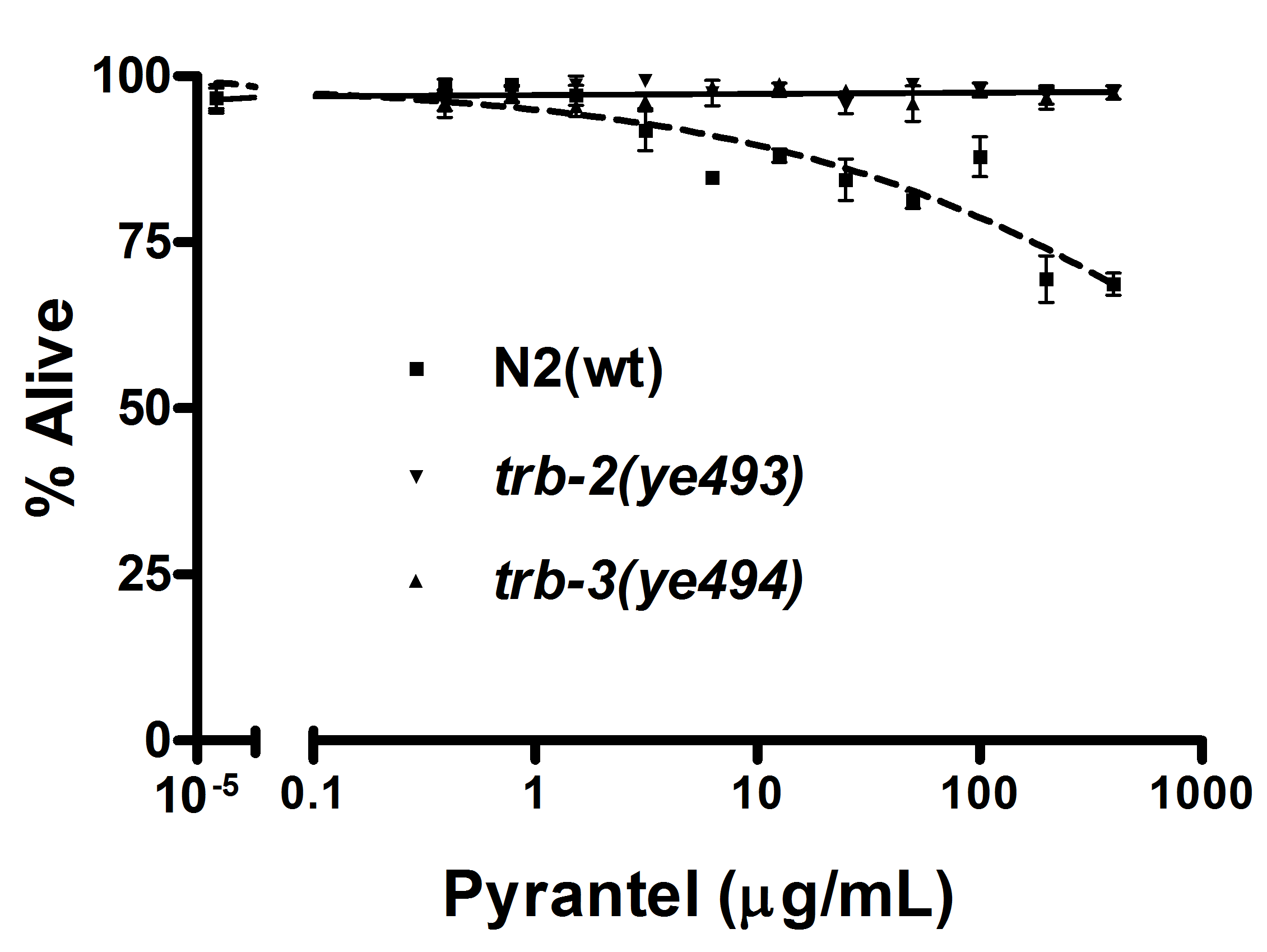

Supplement: Figure S3 — trb-2 and trb-3 mutant animals are resistant to pyrantel. Standard mortality assays were carried out for wild-type (wt) N2 and trb-2 and trb-3 mutant animals as described in the main text. The data come from three replicate experiments with an average of 180 animals per data point. * = P value relative to N2<0.05; ** = P value relative to N2<0.01; *** = P value relative to N2<0.001 (ANOVA analysis, Tukey's HSD test). Error bars represent standard error of the mean. (0.11 MB TIF) [file pntd.0000499.s003.tif]

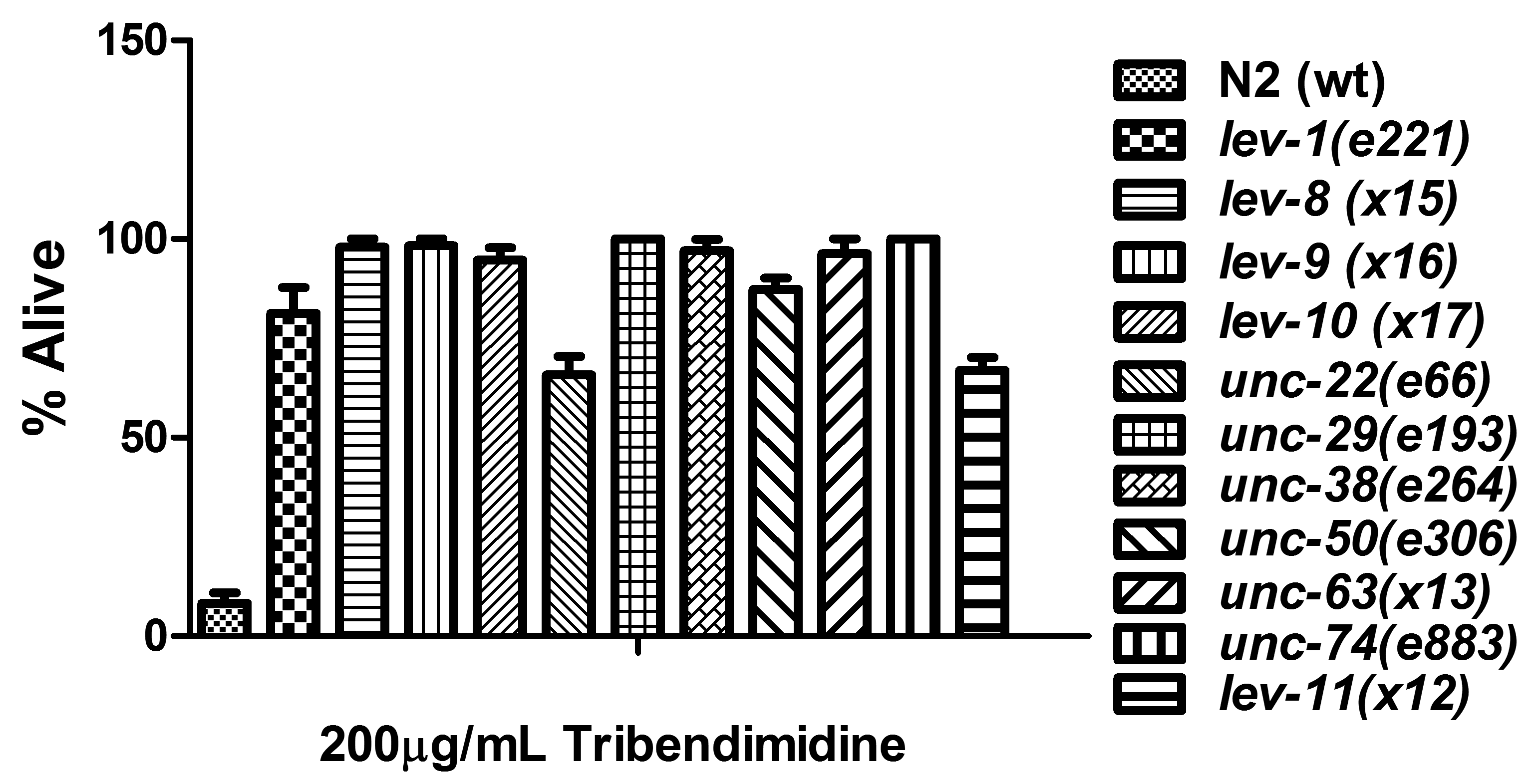

Supplement: Figure S4 — Quantitative resistance of levamisole-resistant mutants at 200 µg/mL tribendimidine. Data are taken from the 200 µg/mL dose in Figure 8. (0.18 MB TIF) [file pntd.0000499.s004.tif]

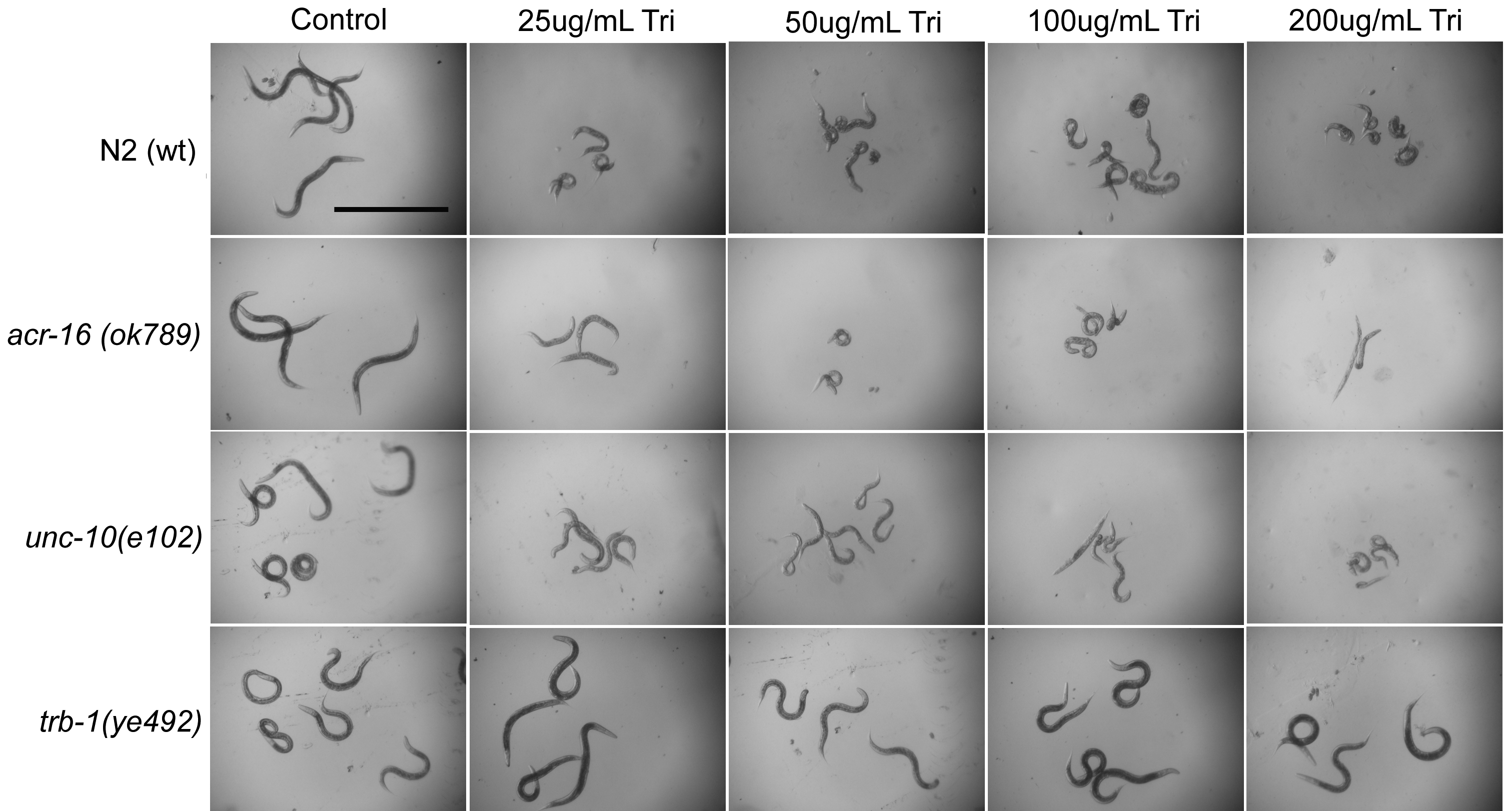

Supplement: Figure S5 — Semi-quantitative analysis of various mutants on tribendimidine (Tri). L4 staged animals of the indicated genotype were placed in wells with the indicated amount of tribendimidine and incubated for 24 h at 25°. The acr-16 and unc-10 mutant animals are clearly susceptible to tribendimidine as shown by the fact that they are as paralyzed as wild-type animals by the drug at all concentrations and that they are more pale in color than wild-type animals even at lower drug concentrations. trb-1 mutant animals were included as a resistant control. Scale bar is 1 mm. (4.93 MB TIF) [file pntd.0000499.s005.tif]
